# Enhancement of biohydrogen production rate in *Rhodospirillum rubrum* by a dynamic CO-feeding strategy using dark fermentation

**DOI:** 10.1186/s13068-021-02017-6

**Published:** 2021-08-06

**Authors:** Alberto Rodríguez, Natalia Hernández-Herreros, José L. García, M. Auxiliadora Prieto

**Affiliations:** 1 Interdisciplinary Platform for Sustainable Plastics towards a Circular Economy‐of the Spanish National Research Council (SusPlast‐CSIC), Madrid, Spain; 2 Polymer Biotechnology Group, Department of Plant and Microbial Biotechnology, Biological Research Center, Margarita Salas”-CSIC, 28040 Madrid, Spain; 3 Environmental Biotechnology Group, Department of Plant and Microbial Biotechnology, Biological Research Center, Margarita Salas”-CSIC 28040, Madrid, Spain

**Keywords:** Hydrogen, *Rhodospirillum rubrum*, Syngas, Dark fermentation, Kinetic model

## Abstract

**Background:**

*Rhodospirillum rubrum* is a purple non-sulphur bacterium that produces H_2_ by photofermentation of several organic compounds or by water gas-shift reaction during CO fermentation. Successful strategies for both processes have been developed in light-dependent systems. This work explores a dark fermentation bioprocess for H_2_ production from water using CO as the electron donor.

**Results:**

The study of the influence of the stirring and the initial CO partial pressure (*p*_CO_) demonstrated that the process was inhibited at *p*_CO_ of 1.00 atm. Optimal *p*_CO_ value was established in 0.60 atm. CO dose adaptation to bacterial growth in fed-batch fermentations increased the global rate of H_2_ production, yielding 27.2 mmol H_2_ l^−1^ h^−1^ and reduced by 50% the operation time. A kinetic model was proposed to describe the evolution of the molecular species involved in gas and liquid phases in a wide range of *p*_CO_ conditions from 0.10 to 1.00 atm.

**Conclusions:**

Dark fermentation in *R. rubrum* expands the ways to produce biohydrogen from CO. This work optimizes this bioprocess at lab-bioreactor scale studying the influence of the stirring speed, the initial CO partial pressure and the operation in batch and fed-batch regimes. Dynamic CO supply adapted to the biomass growth enhances the productivity reached in darkness by other strategies described in the literature, being similar to that obtained under light continuous syngas fermentations. The kinetic model proposed describes all the conditions tested.

## Background

The development of biofuels has multiplied global energy alternatives, aiming the reduction of conventional fossil fuels dependence, carbon footprint and increasing the sustainability of human activities in a context of climate change and depletion of non-renewable resources in the global ecosystem [1]. Global biofuel production reached a record of 154 billion litres in 2018, growing a 7% year-on-year since 2013, and the future prospects point to an increase of 25% in 2024 compared to the previous 7 years [2]. One of the main alternatives in this field is the use of hydrogen as a substitute of hydrocarbons in internal combustion engines or in the production of electricity in fuel cells [3].

Hydrogen (H_2_) is considered one of the most interesting fuels due to its high calorific value (131 MJ/kg), which is 2.6 and 6 times higher than gasoline and methanol, respectively, and the absence of CO_2_ emissions during its combustion [4, 5]. Conventionally, hydrogen come from reforming stages of fossil fuels, covering 93% of the global demand of this compound in 2019 [5]. Hydrogen is also produced from water electrolysis (4% of demand) and biotechnologically, so called biohydrogen (bio-H_2_), from the fermentation of CO_2_, sugars or volatile fatty acids (VFAs) by algae, bacteria or archaea in presence or absence of light [6].

The current H_2_ production methods pursue the reduction of the carbon footprint through the use of solar renewable energy in photovoltaic cells for water electrolysis (green hydrogen) and waste valorization, introducing new alternatives of management, where the biotechnological conversion plays a fundamental role [7]. Bio-H_2_ production has gained much attention due to its sustainability nature and less energy consumptive than physicochemical methods [6]. Moreover, dark fermentation is considered the most appropriate way, as it does not need any external energy, and reaches higher production rates over other procedures [5, 7]. With respect to other biofuels, bio-H_2_ has competitive advantages, such as the absence of carbon dioxide, other greenhouse gasses and pollutant particles upon combustion [8]. Hydrogen is also an energy carrier unlike the rest of carbon-based fuels, and represents the most abundant and lightest reactive gas, which is economical to produce and manufacture if specific routes are employed [8].

*Rhodospirillum rubrum* is a Gram-negative purple non-sulphur bacterium (PNSB), able to produce H_2_ by two different pathways, i.e., photofermentation of organic acids and carbohydrates, and via a water gas-shift reaction from CO, a component of syngas (CO, CO_2_, H_2_) [9]. In the first pathway, light energy is essential to overcome the thermodynamic impediment of converting organic acids into H_2_ [7]. H_2_ is released as a secondary product of nitrogen fixation catalysed by a nitrogenase [10]. During the water gas-shift reaction, CO_2_ and H_2_ are produced because of water reduction by CO (Eq. 1):

1 $${\text{CO}} + {\text{H}}_{2} {\text{O}} \to {\text{CO}}_{2} + {\text{H}}_{2} .$$

This reaction is thermodynamically favourable (Δ*G*^0^ =  − 20 kJ/mol) and requires the simultaneous activity of two enzymes, i.e., a carbon monoxide dehydrogenase (CODH) and a hydrogenase [11]. In addition, ferredoxins (Fd) act as electron transporters [12, 13]. The produced CO_2_ can be fixed by the Calvin–Benson–Bassham cycle (CBB) under photoautotrophic conditions [14]. More recently, the tricarboxylic acid cycle (TCA) and the ethylmalonyl-CoA cycle (EMCoA) have been identified as additional CO_2_ fixation routes in this bacterium [15, 16]. Furthermore, *R. rubrum* is able to accumulate polyhydroxybutyrate (PHB) using syngas [16], sugars (e.g., fructose) and volatile fatty acids (VFAs) (e.g., formate, acetate, propionate, butyrate) as carbon sources [17–19]. From a techno-economic point of view, it has been demonstrated that syngas fermentation of *R. rubrum* is economically viable and technically feasible. The cost of producing the PHA via syngas fermentation is less expensive than producing PHA by sugar fermentation [20]. However, the operating cost of the biorefinery is heavily subsidized by the production and sale of the hydrogen gas, which has been counted as a co-product using *R. rubrum* [20]. Thus, guiding its metabolism towards the co-production of bio-H_2_ is of great interest.

The importance of PNSB as biocatalysts for H_2_ production is also based on their versatility to use VFAs and syngas, both derived from complex organic waste, as carbon and energy sources [21]. These transformations occur under mild operational conditions, i.e., temperatures around 30–40 °C and at atmospheric pressure, while the selectivity towards the desired products increases with respect to the chemical conventional processes [11]. In the case of syngas, the main fermentation challenge is to increase the transformation rate, limited by CO toxicity and its competition, in terms of affinity, by the active sites of the enzymes, with O_2_ and CO_2_ [21].

Most processes described so far that use *R. rubrum* to produce H_2_ from CO are performed under light conditions where an additional carbon source is used as the growth-limiting substrate, mainly acetate, which renders the highest biomass concentration [14]. The influence of CO transfer rate (COTR) by increasing the stirring or gas flow rate has been widely studied, and the optimal operational conditions yielded high H_2_ production rates in continuous regime (32 mmol H_2_ l^−1^ h^−1^) [22].

In absence of light, CO is the growth-limiting substrate, but showed inhibition when the CO ratio exceeded 50% of headspace in closed shaken bottles [12]. In this condition, although the cells were able to use 70% of the initial CO, only 12% of the acetate was consumed at the end of the fermentation, yielding a low amount of biomass. In order to improve this process, we have demonstrated that *R. rubrum* does not require yeast extract for growing in the presence of CO and acetate [16]. Nevertheless, *R. rubrum* requires acetate to grow efficiently using CO as a sole carbon and energy source [16]. Recently, *R. rubrum* has been cultured at lab-bioreactor scale to produce PHB from syngas [15]. A combined C–P nutrient stress enhances the PHB cell content up to a 30% w w^−1^, with a productivity 5 times higher than the C-limited condition, operating in fed-batch with acetate [15]. In both cases, the average H_2_ production rate was around 11 mmol H_2_ l^−1^ h^−1^, feeding a syngas mixture with a 25% of CO [15].

Here, we show the optimal range of conditions necessary to produce H_2_ and PHB with *R. rubrum* at lab-bioreactor scale using CO in darkness, operating in a batch culture. Moreover, using a fed-batch regime by adapting the CO dose to bacterial growth, we have been able to increase the growth rate, yielding 27.2 mmol H_2_ l^−1^ h^−1^ and reducing the operational time in 9 days. Finally, an empirical kinetic model has been proposed, capable to describe the evolution of every condition tested, both in batch or fed-batch operation modes. Our results improve the efficiency of CO into H_2_ by dark fermentation with *R. rubrum* in bioreactor and represent a starting point to explore further applications, such as the creation of “bio-H_2_ fuel cells”, fed only with CO/N_2_ mixtures, operating with cultures of this microorganism in resting cells.

## Results and discussion

### Study of the optimal bioreactor stirring speed to produce PHB and H_***2***_ in a batch culture

The effect of stirring speed in batch cultures of *R. rubrum* is shown in Table 1. Enhancing CO transfer rate by stirring has not a positive effect over the bacterial growth, but it influences significantly the consumption of acetate and the final PHB production. The most remarkable results were obtained when we analysed the PHB and H_2_ yields with respect to CO concentration. Regardless of the selected speed stirring condition, the CO performance of these products remains constant, except in the case of PHB, where the yield decreased when the stirring speed is increased [22]. The tendency was also observed in the values of H_2_ productivity, considering the confidence intervals presented in Table 1.

**Table 1:** Experimental growth parameters, PHB cell content, product yields and productivities by using different stirring speed

Variable	Stirring speed (rpm)			
	250	400	600	1000
*µ*_max_ (h^−1^)	0.057 ± 0.005	0.056 ± 0.006	0.055 ± 0.005	0.057 ± 0.007
*C*_X_ (g l^−1^)	1.46 ± 0.08	1.51 ± 0.06	1.57 ± 0.15	1.65 ± 0.11
PHB (% w w^−1^)	30.4 ± 2.4	25.7 ± 1.7	18.5 ± 1.6	17.1 ± 1.5
Batch time (h)	140 ± 5	134 ± 6	101 ± 4	94 ± 3
*Y*_X/CO_ (g g^−1^)	0.021 ± 0.004	0.022 ± 0.005	0.019 ± 0.002	0.023 ± 0.007
*Y*_X/AC_ (g g^−1^)	1.57 ± 0.33	1.48 ± 0.21	1.29 ± 0.26	1.03 ± 0.19
*Y*_PHB/CO_ (g g^−1^)	0.008 ± 0.0011	0.007 ± 0.0023	0.003 ± 0.0007	0.001 ± 0.0004
*Y*_PHB/AC_ (g g^−1^)	0.84 ± 0.08	0.78 ± 0.05	0.18 ± 0.01	0.13 ± 0.02
$Y_{{{\text{H}}_{2} /{\text{CO}}}}$ (g g^−1^)	0.071 ± 0.006	0.069 ± 0.005	0.07 ± 0.008	0.067 ± 0.007
$Y_{{{\text{CO}}_{2} /{\text{CO}}}}$ (g g^−1^)	1.01 ± 0.11	0.99 ± 0.14	1.03 ± 0.16	1.02 ± 0.24
*P*_PHB_ × 10^3^ (g l^−1^ h^−1^)	3.68 ± 0.18	3.09 ± 0.15	2.31 ± 0.14	1.08 ± 0.10
$P_{{{\text{H}}_{2} }}$ (g l^−1^ h^−1^)	0.038 ± 0.005	0.036 ± 0.004	0.031 ± 0.006	0.033 ± 0.008

The availability of CO has been reported as a limitation of H_2_ and PHB production in this process, due to the toxicity of high levels of dissolved CO (*DCO*) in *R. rubrum* [22, 23]. The stirring speed increases the COTR by the volumetric mass transfer, k_L_a. However, this strategy causes cellular damage due to either a shear/hydrodynamic stress or an excess of the nutrient supply from the gas to the liquid phase [24, 25]. Surprisingly, *R. rubrum* cells experienced a great resistance to shear stress under aggressive stirring conditions (see data of runs conducted at 600 and 1000 rpm in Table 1). This constitutes a novel and interesting response, since in other bioprocesses influenced by the gas–liquid mass transfer rate, the negative effect of hydrodynamic stress is observed in a similar range of stirring speed conditions studied in this work [26]. From these analyses, a stirring speed of 250 rpm was selected as the optimal value for further experiments.

### Study of the initial CO partial pressure (*p*_CO_) to produce PHB and H_2_ in a batch culture

To determine the influence of the initial concentration of CO in the bioreactor, seven batch runs were performed, employing different *p*_CO_ conditions: 0.10, 0.20, 0.40, 0.50, 0.60, 0.75 and 1.00 atm. For each culture the final broth composition, regarding acetate concentration, biomass produced, specific growth rate, CO consumed and H_2_–CO_2_ produced are shown in Table 2. The most remarkable changes occurred when the *p*_CO_ was increased above 0.20 atm, where the acetate is completely consumed, the bacterial growth rate reached the highest value (around 0.06 h^−1^), yielding the maximum PHB accumulation (25–30% w w^−1^). In the gas phase, the water gas-shift reaction was unbalanced, which means that there was an important fraction of CO_2_ incorporated into cell metabolism (33–40%). This ratio was maintained proving the ability of *R. rubrum* to assimilate CO_2_ in darkness [15, 16]. The process was inhibited at *p*_CO_ of 1.00 atm, according to the values of the growth rate and the batch time, although the cells were capable to consume high amounts of CO, yielding more biomass (Table 2).

**Table 2:** Final broth and outlet gas composition in batch fermentations under several initial *p*_CO_ conditions

*p*_CO_ (atm)	Acetate	CO	Total biomass		PHB		Gases produced	
	Conversion (%)	Total uptake (mol)	*µ* (h^−1^)	*C*_X_ (g l^−1^)	Cell content (% w w^−1^)	Titre (g l^−1^)	H_2_ (mol)	CO_2_ (mol)
0.10	21.1 ± 5.2	1.05 ± 0.12	0.049 ± 0.002	0.36 ± 0.04	10.8 ± 2.1	0.04 ± 0.01	1.05 ± 0.05	0.99 ± 0.09
0.20	37.4 ± 4.3	1.89 ± 0.24	0.058 ± 0.006	0.86 ± 0.09	14.8 ± 0.8	0.13 ± 0.01	1.81 ± 0.16	1.11 ± 0.11
0.40	100 ± 11.1	2.71 ± 0.26	0.058 ± 0.004	1.32 ± 0.11	26.5 ± 4.2	0.31 ± 0.01	2.48 ± 0.27	1.57 ± 0.17
0.50	100 ± 5.2	2.61 ± 0.67	0.057 ± 0.005	1.46 ± 0.16	30.4 ± 2.4	0.35 ± 0.03	2.59 ± 0.13	1.67 ± 0.07
0.60	100 ± 2.2	4.57 ± 0.41	0.063 ± 0.008	1.74 ± 0.21	24.1 ± 3.6	0.38 ± 0.06	4.55 ± 0.36	2.76 ± 0.22
0.75	100 ± 2.7	4.06 ± 0.71	0.061 ± 0.007	1.63 ± 0.24	24.8 ± 2.3	0.35 ± 0.04	3.96 ± 0.41	2.50 ± 0.25
1.00	100 ± 14.2	4.94 ± 2.41	0.040 ± 0.005	2.06 ± 0.54	17.4 ± 3.3	0.32 ± 0.09	4.88 ± 0.54	3.22 ± 0.35

**Fig. 1:**
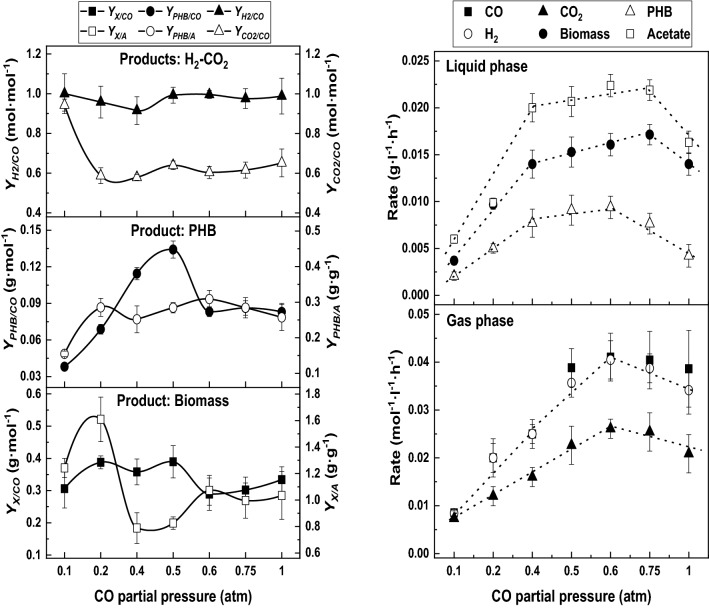
Product yields (left) and maximum production or uptake rates (right) under different initial *p*_CO_ conditions

This tendency was also confirmed in the product yields values, shown in Fig. 1A–C. An equimolar 1:1 ratio was maintained in all the range studied for H_2_ (Fig. 1A, up to the left). The evolution of $Y_{{{\text{CO}}_{2} /{\text{CO}}}}$ reinforces the idea of CO_2_ assimilation by *R. rubrum*: if initial *p*_CO_ was higher than 0.1 atm, the yield decreases from 1 to 0.6 mol mol^−1^, which means that around 40% of this compound produced via water gas-shift reaction is being assimilated by the cells. PHB yields with respect to acetate and CO concentrations followed parallel tendencies (Fig. 1B, middle left). If the initial CO molar fraction increases, *Y*_PHB/A_ reached a steady value around 0.30–0.35 g g^−1^. However, *Y*_PHB/CO_ increased firstly up to a *p*_CO_ of 0.5 atm to further decrease and remain stable at 0.085 g mol^−1^. These results suggested that PHB is synthesized mainly from acetate at *p*_CO_ larger than 0.5 atm. Biomass yields with respect to the carbon sources evolved differently depending if the cells use CO or acetate (Fig. 1C, down left). In the first range of *p*_CO_ conditions (i.e., 0.1–0.5 atm), *Y*_X/CO_ value moved from 0.35 to 0.40 g mol^−1^, while at higher *DCO* concentrations the *Y*_X/CO_ value decreases slightly to 0.30 g mol^−1^. In the case of *Y*_X/A_, the changes were observed at lower *DCO* (i.e., *p*_CO_ > 0.20 atm) and the decrease was very deep. This observation suggests a change in carbon source to produce biomass, i.e., CO_2_ started to be assimilated and the redirection of the acetate flow to PHB production (Fig. 1C).

The maximum values of production or consumption rates of the molecular species involved in the synthesis of PHB and H_2_ from syngas by *R. rubrum* are presented in Fig. 1D, E. The first section (Fig. 1D, up to the right) corresponds to the dynamics of the liquid phase. Three different regions can be defined with respect to *p*_CO_ condition. Firstly, from 0.1 to 0.4 atm of CO their values increase with CO pressure. Secondly, we observe an intermediate region from 0.5 to 0.75 atm of *p*_CO_, where these rates reach their maximum value. Finally, when *p*_CO_ is higher than 0.75 atm we observed an inhibition by an excess of CO causing a significant decrease in the values of PHB, biomass and acetate. In the case of the gas phase (Fig. 1E, down right), two different trends have been identified with respect to CO feeding: from 0.1 to 0.6 atm of CO, every single rate increases with *p*_CO_, while if this variable is higher, an appreciable decrease of water gas-shift reaction global rate has been observed.

**Fig. 2:**
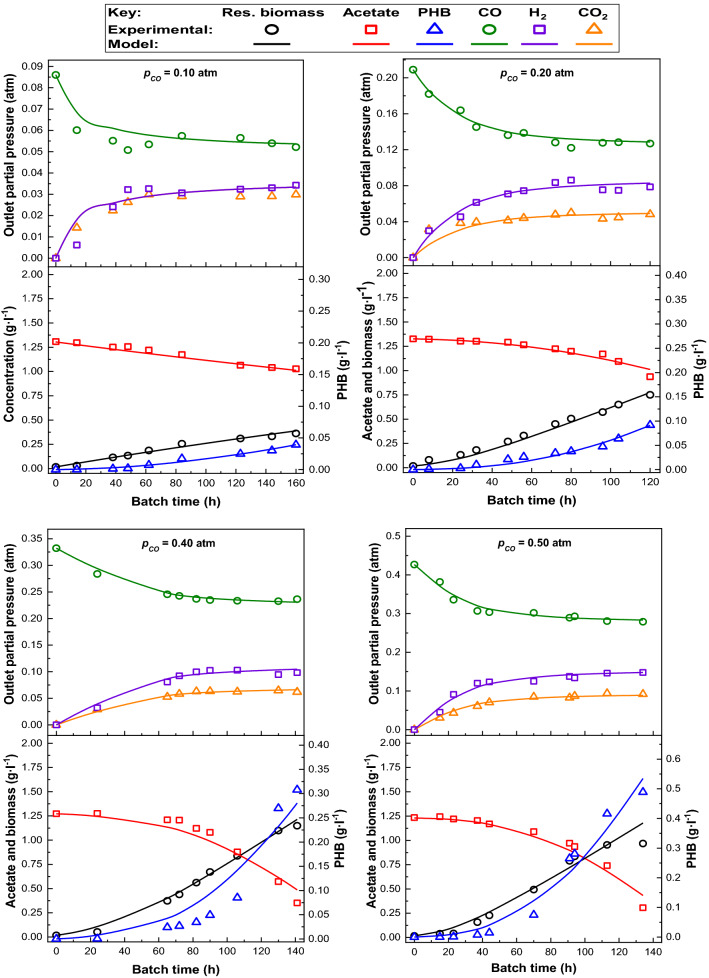
Experimental and predicted data provided by the kinetic model under several initial *p*_CO_ (0.10–0.50 atm)

From these observations a *p*_CO_ of 0.60 atm was selected as the optimal to cultivate *R. rubrum* in darkness. It provided the highest specific growth rate (0.063 h^−1^) and reached the maximum PHB production (around 26% w w^−1^) releasing 4.55 mol of H_2_, which was the most promising result if the consuming time for batch process is considered (117 vs. 222 h, see below). Furthermore, under this CO concentration the acetate consumption rate was the highest observed in this study (less than 5 days were needed for a complete depletion, see above Fig. 2E).

### Kinetic model

Kinetic and statistical model parameters values for the runs performed with different initial CO concentrations are shown in Table 3. Their values reflect the tendencies observed in the culture when *p*_CO_ was increased, even in the gas or the liquid phase. Considering the growth, the kinetic constants do not present significant changes, showing their dependence on CO_2_ availability in the medium. This performance matches with the values of the kinetic of CO_2_ yield with respect to biomass (*Ʋ*$_{{{\text{CO}}_{2} /{\text{X}}}}$). For the PHB production constant (*K*_P_), the positive effect in the accumulation of this biopolymer when the CO availability was increased is also reflected in its values. This evolution was similar to that followed by the parameters involved in the water gas-shift reaction.

**Table 3:** Kinetic and statistical parameters of the proposed model

*p*_CO_ (atm)	Kinetic parameter							
	*K*_X, A_ × 10^3^	$K_{{{\text{X}},{\text{ CO}}_{2} }}$	*K*_1_	*K*_2_ × 10^3^	*K*_P_ × 10^3^	*Ʋ*_A/P_	*Ʋ*_A/X_	*Ʋ*$_{{{\text{CO}}_{2} /{\text{X}}}}$
	(g X g A^−1^ h^−1^)	(g X mol CO_2_^−1^ h^−1^)	(mol H_2_ g X^−1^ h^−1^)	(mol CO g X^−1^)	(g PHB g X^−1^ h^−1^)	(g A g PHB^−1^)	(g A g X^−1^)	(g CO_2_ g X^−1^)
0.10	2.01 ± 0.51	–	0.13 ± 0.04	0.65 ± 0.21	1.16 ± 0.39	0.25 ± 0.04	0.76 ± 0.03	–
0.20	–	5.31 ± 0.51	0.26 ± 0.03	1.39 ± 0.16	2.18 ± 0.74	2.67 ± 0.88	0.12 ± 0.04	1.04 ± 0.17
0.40	–	5.76 ± 0.76	0.18 ± 0.02	1.31 ± 0.64	3.95 ± 0.74	2.66 ± 0.98	0.09 ± 0.03	0.82 ± 0.11
0.50	–	4.08 ± 0.54	0.34 ± 0.04	2.25 ± 0.29	7.79 ± 2.29	1.55 ± 0.45	0.04 ± 0.02	1.31 ± 0.11
0.60	–	5.18 ± 0.51	0.21 ± 0.01	1.51 ± 0.08	5.92 ± 0.95	2.64 ± 0.31	0.26 ± 0.03	0.87 ± 0.07
0.75	–	5.18 ± 0.54	0.18 ± 0.02	1.72 ± 0.16	6.03 ± 0.99	1,83 ± 0.32	0.52 ± 0.02	0.85 ± 0.14
1.00	–	7.74 ± 0.76	0.08 ± 0.01	1.27 ± 0.33	1.52 ± 0.11	2.69 ± 0.53	0.18 ± 0.04	0.65 ± 0.31

*p*_CO_ (atm)	Statistical parameter							
	*F*_calc_	*F*_tab_	RMSE	SSR	VE (%)			
0.10	1529	2.249	0.002	2 × 10^–4^	91.7			
0.20	4399	2.249	0.003	6 × 10^–4^	96.6			
0.40	9066	2.249	0.003	5 × 10^–4^	98.1			
0.50	6314	2.249	0.005	1 × 10^–3^	97.2			
0.60	14,002	2.346	0.003	4 × 10^–4^	99.3			
0.75	24,872	2.249	0.003	6 × 10^–4^	99.1			
1.00	4426	2249	0.012	7 × 10^–3^	90.1			

On the other hand, the kinetic yields of the conversion of acetate into PHB oscillate between 1.5 and 2.7 (note the carbon ratio 2:4 between acetate and 3-hydroxybutyrate). These values determine the distribution of this substrate with respect to biomass (*Ʋ*_A/X_). According to the experimental observations of CO_2_ production rate (see also Fig. 1E), the kinetic yields of this compound with respect to biomass were maintained in a short range of values, which can suggest that the amount of this compound that is incorporated into the biomass was not dependent of the dissolved CO available for the cells.

All the fittings satisfy the statistical criteria explained below, reinforcing the validity of the proposed kinetic model: *F*-test, the root medium squared error (RMSE) and the variation explained (VE) reinforced the validity of the proposed kinetic model.

**Fig. 3:**
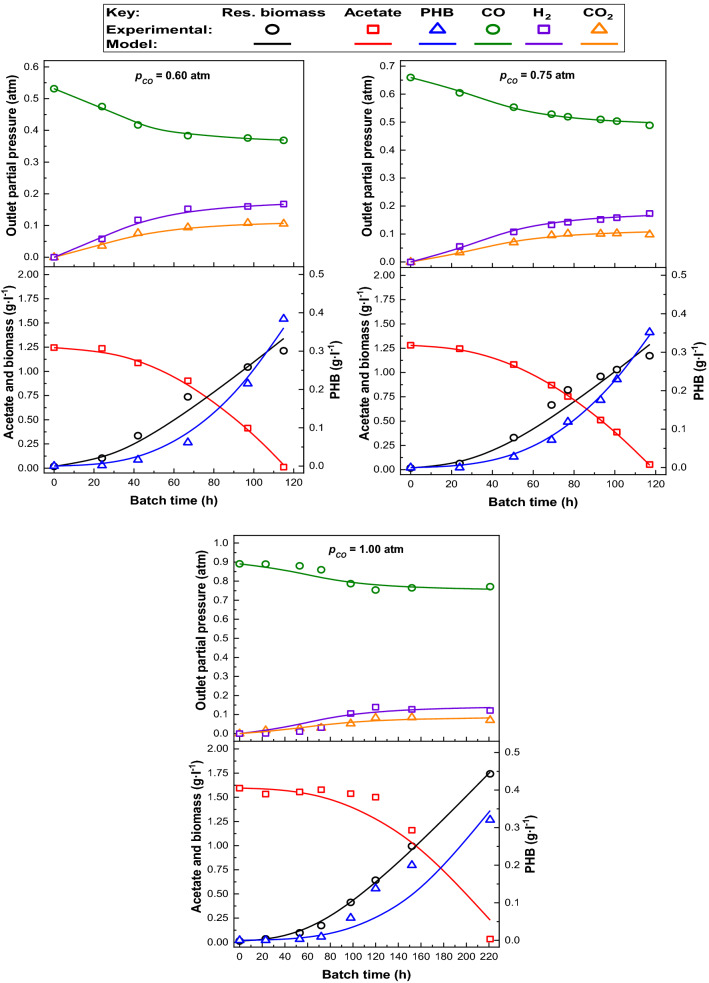
Experimental and predicted data provided by the kinetic model under several initial *p*_CO_ (0.60–1.00 atm)

Experimental data and kinetic model predictions are presented in Figs. 2 and 3. The good fitting of the model is clearly observed, being able to reproduce the batch fermentation results. It is, therefore, a valid and robust approach to simulate the dynamics of this bioprocess in a wide range of operational conditions.

### Fed-batch experiments under different CO-feeding strategies

**Fig. 4:**
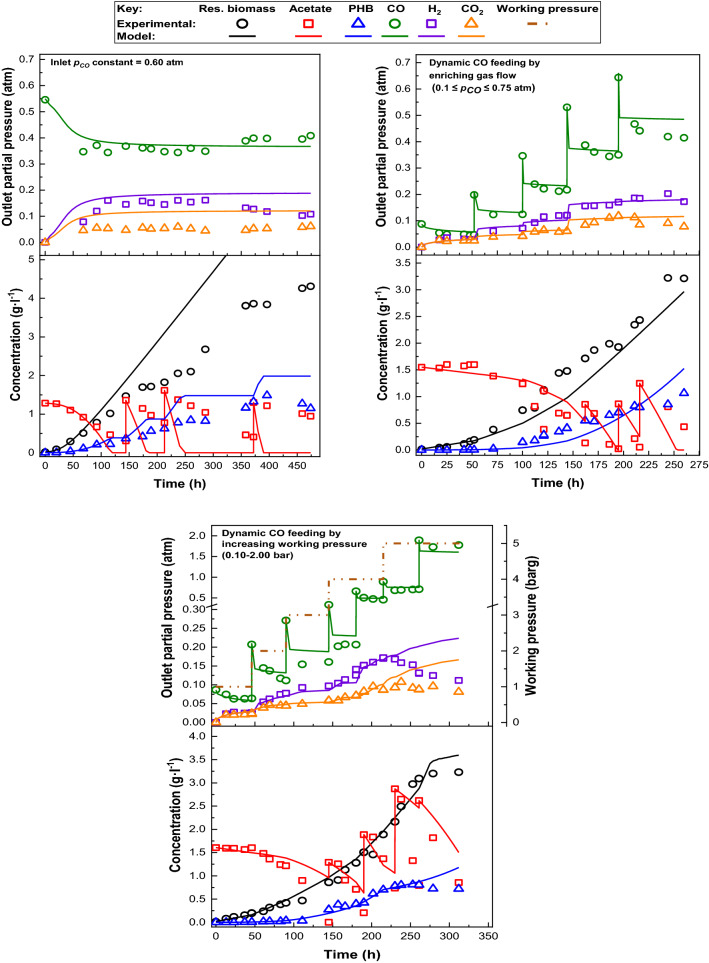
Experimental and predicted data provided by the kinetic model in fed-batch applying different CO-feeding strategies

The experimental results of the fed-batch runs are presented in Table 4. Furthermore, the evolution with time of the molecular species involved in gas and liquid phases together with the predicted curves given by the kinetic model are included in Fig. 4. Firstly, the optimal condition established in the previous section resulted inadequate in fed-batch regime (Fig. 4A, up to the left). After the first pulse of acetate, the experimental measurements clearly deviated from the predictions provided by the kinetic model. In this condition we reached the highest PHB and total biomass concentrations, whereas the COUR and $R_{{{\text{H}}_{2} }}$ average values were similar to the achieved in batch regime (Fig. 1E), but around 20 days were needed to reach these figures. Therefore, two additional alternatives for operating in fed-batch regimes were explored, subsequently based on developing a dynamic CO supply to *R. rubrum* cultures, adapting the CO dose to the growth for avoiding CO toxicity and improving the use of the key substrates (CO and acetate), while maintaining a high H_2_ production. According to the data shown in Table 4 and Fig. 4B, C, both dynamic CO-feeding strategies were beneficial to maintain the maximum growth rate. Moreover, the use of acetate was enhanced from 76 to 83% or 91%, and the operation time was clearly reduced from 20 to 11 days or 13 days, respectively (Table 4). According to Table 5, these procedures increased H_2_ production rates when compared to previous works operating in fed-batch with the same culture medium in darkness. In fact, our results were very close to those achieved with *R. rubrum* in continuous light cultures and other PNSB strains, with high H_2_ productivities by photofermentation of VFA’s and sugars (Table 5). Furthermore, our bio-H_2_ productivity in fed-batch regime is comparable to others achieved with thermophilic microorganisms, such as *Thermoanaerobacterium thermosaccharolyticum* (25.9 mmol l^−1^ h^−1^) [27] or *Thermotoga maritima* (28 mmol l^−1^ h^−1^) [28].

**Table 4:** Experimental results of the fed-batch runs performed under several CO-feeding strategies

CO-feeding strategy	Operation time (days)	Liquid phase			Gas phase		
		Total *C*_X_ (g l^−1^)	PHB (%) (w w^−1^)	Acetate conversion (%)	Avg. production–consumption rates (mmol l^−1^ h^−1^)		
					CO	H_2_	CO_2_
Constant (*p*_CO_ = 0.60 atm)	19.8	5.35	27.6	76.6	39.2	32.3	13.4
Dynamic (from 0.1 to 0.75 atm, enriching gas flow)	10.8	4.28	24.9	91.4	29.1	27.2	15.3
Dynamic (from 0.1 to 2 atm, increasing working pressure)	13	3.95	20.8	83.2	24.3	25.1	15.4

**Table 5:** Comparative hydrogen production rate ($R_{{{\text{H}}_{2} }}$) in PNSB under photosynthetic or dark fermentation conditions

Strain	Operation	Metabolic pathway	Carbon sources	$R_{{{\text{H}}_{2} }}$ mmol l^−1^ h^−1^	References
*Rhodopseudomonas palustris*	Batch	Photofermentation	Malate + glutamate	0.3	[43]
	Batch	Photofermentation	Lactate	2.2	[44]
*Rhodobacter sphaeroides*	Batch	Photofermentation	Acetate	0.9	[36]
	Batch	Photofermentation	Acetate + butyrate + glutamate	2.1	[45]
	Batch	Photofermentation	Acetate + butyrate + glutamate	6.1	[46]
*Rhodobacter capsulatus*	Batch	Photofermentation	Glucose	32	[47]
	Batch	Photofermentation	Acetate	0.3	[48]
	Batch	Photofermentation	Acetate + butyrate	0.2	[49]
	Batch	Photofermentation	Acetate + glutamate	1	[50]
*Rhodospirillum rubrum*	Batch (light)	Syngas fermentation	Acetate + syngas	0.2	[13]
	Continuous (light)	Syngas fermentation	Acetate + syngas	32	[22]
	Fed-batch (dark)	Syngas fermentation	Acetate + syngas	11	[15]
	Fed-batch (dark)	Syngas fermentation	Acetate + syngas	27	This work
	Continuous	Photofermentation	Lactate + glutamate	1	[51]

The unique capability of *R. rubrum* to produce H_2_ and PHB from syngas and VFA’s can be improved if a doubled C–P nutrient limitation is induced, reaching a highest biopolymer accumulation of 30% w w^−1^ [15]. Monomer composition has been modified by using engineered strains containing genes of *Pseudomonas putida* or *Cupriavidus necator* (formerly *Ralstonia eutropha*) to produce co-polymers of 3-hydroxyoctanoate and 3-hydroxydecanoate or 3-hydroxybutyrate and 3-hydroxyvalerate, respectively [29, 30]. In both cases the final PHA content was lower than the content achieved with the wild type strain in this work (7.1% and 14.8% w w^−1^, respectively) [29, 30]. Other microorganisms can use syngas as carbon source, but they do not produce PHA naturally, such as clostridia [31]. However, by using recombinant strains of *Clostridium coskatii*, PHB was synthesized heterotrophically (3.4% w w^−1^) and autotrophically (1.12 w w^−1^) [32]. PHA producers were able to use the CO_2_ and H_2_ contained in syngas, but CO can only be catabolized when the *cox* subcluster found in some carboxydotrophic strains is heterologously expressed [31]. In this sense, promising results have been achieved in closed shaken bottles by introducing the *cox* genes of *Oligotropha carboxidovorans* in *C. necator* H16, achieving a final PHB cell content around 50% w w^−1^ [33].

Adapting the dose of CO to bacterial growth provided *R. rubrum* a greater tolerance to this compound (Fig. 4C). In the later stage of the run, the cells remain in exponential growth phase, even when the culture was subjected to 1 bar of CO, a condition that was inhibitory in batch mode. This observation is interesting for further developing new strategies for H_2_ production from CO with this strain, applying the current CO dynamic dose in fed-batch to concentrate the culture in biomass and evaluating the H_2_ production capability without additional carbon sources (i.e., acetate).

## Conclusions

The capability of co-producing H_2_ and PHB from CO under anaerobic conditions is unique in *R. rubrum* and therefore the use of this strain to produce H_2_ from CO by a dark fermentation has been evaluated at bioreactor scale. We have analysed the influence of the stirring speed, the initial CO partial pressure and the operation in batch and fed-batch regimes in order to optimize the production of H_2_. We have demonstrated that adapting the CO feeding to growth enhances the productivity reached in darkness by other strategies described so far, being similar to that obtained under light continuous syngas fermentations in other PNSB, yielding 27.2 mmol H_2_ l^−1^ h^−1^. The kinetic model proposed was able to describe the experimental results obtained, even in batch or fed-batch regimes. Our results will pave the way to increase the production of bio-H_2_ that as commented above still represent a low fraction of the total H_2_ market. Moreover, this process allows the utilization of CO not only from conventional origins, such as gasification or steel mills, but also from most advanced processes such CO_2_ electrolysis or CO_2_ and water co-electrolysis.

The high efficiency of the water gas-shift reaction described in this study opens the possibility of exploring this transformation using different operational modes, for instance using resting cells instead of growing cells, whose format and simplicity can be exported to the creation of new fuel cells, producing bio-H_2_ from CO in a continuous operation.

## Methods

### Bacterial strain, growth conditions and media

*R. rubrum* S1 (ATCC 11170) was the strain used in this work. The strain was stored at − 80 °C in a 50% glycerol–saline serum solution closed vials. Before bioreactor inoculation, pre-cultures of this microorganism were grown in closed serum bottles inoculated with the frozen stock under anaerobic conditions (CO/N_2_, 50/50 mol mol^−1^) on RRNCO medium (initial pH = 7.0) [12] supplemented with 15 mM acetate at 30 °C and 200 rpm until the culture reaches the stationary phase (i.e., OD_600_ 1.2–1.5). The RRNCO medium composition and the protocol followed to prepare the serum bottles have been described previously [16]. The initial optical density was fixed at 0.05 for both shaken bottles and bioreactors [15, 16]. The pre-inoculum stage lasted between 7 and 10 days.

### Batch experiments in bioreactor: study of the optimal stirring speed and the initial *p*_CO_

**Fig. 5:**
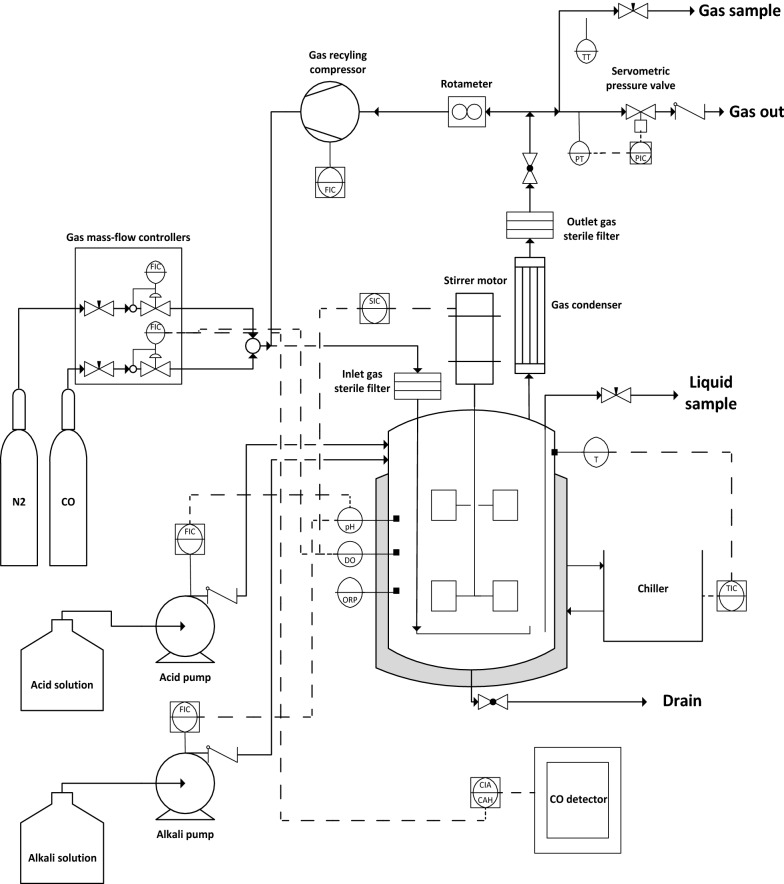
Schematic layout of the tailor-made bioreactor unit for syngas fermentation

The experiments with different initial CO concentrations were carried out in a 2-l stainless steel tailor-made bioreactor. The schematic diagram of the experimental setup is shown in Fig. 5. The gas supply consists of two mass flow controllers (MFCs), represented as “flow indicator controllers” (FIC). The working pressure is controlled by a servometric value in the outlet gas stream (PIC), programmed to work between 1 and 5 barg. The unit is place inside an extractor hood with continuous flux. The CO detector acts over the inlet CO-line through a controller (CIA-CAH), in order to ensure the work safety conditions. The working volume was 1 l and 4% (v v^−1^) inoculum from a stationary phase grown pre-culture, as indicated above [15]. The vessel was sterilized by autoclaving at 121 °C for 20 min before inoculation. The operational conditions are indicated as follows: 30 °C of temperature, 0.1 vvm (100 ml min^−1^) of total CO/N_2_ inlet gas flow and the pH maintained around 7.0–7.2 by adding periodic syringe pulses of 10 M NaOH [22]. For stirring studies, the stirrer speed was modified from 250 to 1000 rpm [14, 34]. Once its optimal value was selected, the influence of initial *p*_CO_ was determined varying the inlet gas stream composition from 0.1 to 1 atm of CO, operating under atmospheric working pressure (1 atm). All runs have been performed by triplicate.

### Fed-batch experiments in bioreactor: adapting CO dose in the gas phase to optimize growth and hydrogen production

Fed-batch experiments were carried out using the same operational conditions of batch runs (i.e., temperature, optimal stirring speed, gas flow, pH and initial biomass concentration). Pulses of acetate were added periodically from 1 M acetate solution. The CO supply in the gas stream was changed by: (i) maintaining the initial *p*_CO_ in a constant value, according to the optimal condition determined in the prior study; (ii) increasing the inlet gas stream in CO according to the bacterial growth from 10 to 75% of the total gas flow (100 ml min^−1^); (iii) enriching the CO content in the culture medium by increasing the working pressure inside the vessel from 1 to 5 barg.

### Analytical methods

#### Biomass quantification and substrate monitoring in the liquid phase

The concentration of biomass (*C*_X_) was measured by optical density at 600 nm (Shimadzu UV–visible spectrophotometer UV-1603, Japan), correlating the sample absorbance (OD_600_) with cell dry biomass (g l^−1^) according to Eq. (2) [35]:

2 $$C_{{\text{X}}} \left( {{\text{g}}\;{\text{L}}^{ - 1} } \right) = 0.3488 \times {\text{OD}}_{600} .$$

Acetate concentration during syngas fermentation was determined by HPLC (Agilent Technologies 1200 Infinity II series, USA), using an Aminex HPX-87H C column (300 × 7.8 mm, Bio-Rad, USA), coupled to a 1260 Infinity II Diode Array and Refractive Index Detectors, working at room temperature. The mobile phase employed was 0.005 M H_2_SO_4_ at flow rate of 0.5 ml min^−1^. Column temperature was controlled at 50 °C and the sample volume was 15 µl [16, 35].

#### Gas analysis

The analytical protocol for H_2_, CO and CO_2_ quantification has been described elsewhere [16]. Gas samples from the headspace in serum bottles or the outlet gas stream in the bioreactor runs were taken from the cultures at different times and transferred to headspace-vials, where a known fraction of Ne (*p*_Ne_ = 0.25 bar), used as the internal gas standard, was added. Calibration curves of H_2_, CO_2_ and CO were performed by representing the ratio of the partial pressures of the compounds and the internal standard (*p*_i_/*p*_Ne_) with respect to the ratio of areas provided by the chromatogram (*A*_i_/*A*_Ne_).

#### Biopolymer quantification by GC/MS analysis

The PHB content was measured by GC/MS determining the fraction of the methanolysed biopolymer by using a described protocol [16]. A standard curve from 0.5 to 2 mg of PHB (Sigma-Aldrich) was used to interpolate sample data [36].

### Mathematical methods

#### CO, H_2_ and CO_2_ mass balances

Mass balances for the six compounds involved in syngas fermentation are shown in Table 6, through Eqs. (10–18). The symbols “k_L_a^i^” are referred to the volumetric mass transfer coefficient for CO, CO_2_ and H_2_, calculated through gas diffusivities [37]. “*K*_H,I_” is the value of Henry’s constant for each compound in water, “*R*” is the ideal gas law constant, “*T*” is the working temperature and “*p*_i_*” is the concentration in equilibrium with the liquid phase [26].

**Table 6:** Simplified reaction network, kinetic equations and mass balances of the kinetic model proposed

Simplified reaction network		Kinetic equations	
Equation	No.	Equation	No.
${\text{CO}} + {\text{H}}_{2} {\text{O}} \to {\text{CO}}_{{2}} + {\text{H}}_{2} ;\; r_{1}$	2	$r_{1} = \frac{{K_{1} \cdot p_{{{\text{CO}}}}^{{{\text{OUT}}}} \cdot C_{{\text{X}}} }}{{K_{2} \cdot K_{{{\text{H}}, {\text{CO}}}} \cdot C_{{\text{X}}} + p_{{{\text{CO}}}}^{{{\text{OUT}}}} }}$	6
$\vartheta_{{{\text{A}}/{\text{PHB}}}} \cdot {\text{A}} \to {\text{PHB}};\; r_{2}$	3	$r_{2} = K_{{\text{P}}} \cdot C_{{\text{X}}}$	7
${\text{If}}\;p_{{{\text{CO}}}}^{{{\text{IN}}}} \le 0.10 \;{\text{atm}}: \vartheta_{{{\text{A}}/{\text{X}}}} \cdot {\text{A}} \to {\text{X}}; r_{3}$	4	$r_{3} = K_{{{\text{X}}, {\text{A}}}} \cdot C_{{\text{A}}}$	8
${\text{If}}\;p_{{{\text{CO}}}}^{{{\text{IN}}}} > 0.10\;{\text{ atm}}: \vartheta_{{{\text{CO}}_{2} /{\text{X}}}} \cdot {\text{CO}}_{2} + \vartheta_{{{\text{A}}/{\text{X}}}} \cdot {\text{A}} \to {\text{X}}; \;r_{4}$	5	$r_{4} = K_{{{\text{X}}, {\text{CO}}_{2} }} \cdot \left( {\frac{{p_{{{\text{CO}}_{{2}} }}^{{{\text{OUT}}}} }}{{K_{{{\text{H}}, {\text{CO}}_{2} }} }}} \right)$	9

Mass balances		
Compound	Equation	No.
CO	$\frac{1}{R \cdot T}\left( {\frac{{{\text{d}}p_{{{\text{CO}}}}^{{{\text{OUT}}}} }}{{{\text{d}}t}}} \right) = \frac{{{\text{k}}_{{\text{L}}} {\text{a}}^{{{\text{CO}}}} }}{{K_{{{\text{H}},{\text{ CO}}}} }} \cdot \left( {p_{{{\text{CO}}}}^{{{\text{IN}}}} - p_{{{\text{CO}}}}^{{{\text{OUT}}}} } \right) - r_{1}$	10
H_2_	$\frac{1}{R \cdot T}\left( {\frac{{{\text{d}}p_{{{\text{H}}_{2} }}^{{{\text{OUT}}}} }}{{{\text{d}}t}}} \right) = - \frac{{{\text{k}}_{{\text{L}}} {\text{a}}^{{{\text{H}}_{2} }} }}{{K_{{{\text{H}}, {\text{H}}_{2} }} }} \cdot \left( {p_{{{\text{H}}_{2} }}^{{{\text{OUT}}}} - p_{{{\text{H}}_{2} }}^{*} } \right) + r_{1}$	11
CO_2_	${\text{If}}\;p_{{{\text{CO}}}}^{{{\text{IN}}}} \le 0.10 \;{\text{atm}}:\frac{1}{R \cdot T}\left( {\frac{{{\text{d}}p_{{{\text{CO}}_{2} }}^{{{\text{OUT}}}} }}{{{\text{d}}t}}} \right) = - \frac{{{\text{k}}_{{\text{L}}} {\text{a}}^{{{\text{CO}}_{2} }} }}{{K_{{{\text{H}}, {\text{CO}}_{2} }} }} \cdot \left( {p_{{{\text{CO}}_{2} }}^{{{\text{OUT}}}} - p_{{{\text{CO}}_{2} }}^{*} } \right) + r_{1}$	12
	${\text{If}}\;p_{{{\text{CO}}}}^{{{\text{IN}}}} > 0.10 \;{\text{atm}}:\frac{1}{R \cdot T}\left( {\frac{{{\text{d}}p_{{{\text{CO}}_{2} }}^{{{\text{OUT}}}} }}{{{\text{d}}t}}} \right) = - \frac{{{\text{k}}_{{\text{L}}} {\text{a}}^{{{\text{CO}}_{2} }} }}{{K_{{{\text{H}}, {\text{CO}}_{2} }} }} \cdot \left( {p_{{{\text{CO}}_{2} }}^{{{\text{OUT}}}} - p_{{{\text{CO}}_{2} }}^{*} } \right) + r_{1} - \vartheta_{{{\text{CO}}_{2} /{\text{X}}}} \cdot r_{4}$	13
Residual biomass (X)	${\text{If}}\;p_{{{\text{CO}}}}^{{{\text{IN}}}} \le 0.10 \;{\text{atm}}: \frac{{{\text{d}}C_{{\text{X}}} }}{{{\text{d}}t}} = r_{3}$	14
	${\text{If}}\;p_{{{\text{CO}}}}^{{{\text{IN}}}} > 0.10\;{\text{ atm}}: \frac{{{\text{d}}C_{{\text{X}}} }}{{{\text{d}}t}} = r_{4}$	15
PHB	$\frac{{{\text{d}}C_{{{\text{PHB}}}} }}{{{\text{d}}t}} = r_{2}$	16
Acetate (A)	${\text{If}}\;p_{{{\text{CO}}}}^{{{\text{IN}}}} \le 0.10\;{\text{ atm}}: \frac{{{\text{d}}C_{{\text{A}}} }}{{{\text{d}}t}} = - \vartheta_{{{\text{A}}/{\text{PHB}}}} \cdot r_{2} - \vartheta_{{{\text{A}}/{\text{X}}}} \cdot r_{3}$	17
	${\text{If}}\;p_{{{\text{CO}}}}^{{{\text{IN}}}} > 0.10\;{\text{ atm}}: \frac{{{\text{d}}C_{{\text{A}}} }}{{{\text{d}}t}} = - \vartheta_{{{\text{A}}/{\text{PHB}}}} \cdot r_{2} - \vartheta_{{{\text{A}}/{\text{X}}}} \cdot r_{4}$	18

#### Product yields, productivities and specific growth rates

The analyses of the experimental results have been done by determining the following parameters: the product yield with respect to the substrate consumed (*Y*_*i*/*j*_, g i g j^−1^), the productivity of the different final products (*P*_*i*_, g i l^−1^ h^−1^) and the specific biomass growth rate in each condition (*µ*, h^−1^) are defined by Eqs. (19–21), respectively:

19 $$Y_{i/j} = \frac{{C_{i, F} - C_{i, 0} }}{{C_{j, 0} - C_{j, F} }},$$

20 $$P_{i} = \frac{{C_{i, F} - C_{i, 0} }}{{t_{F} }},$$

21 $$C_{{\text{X}}} \left( t \right) = C_{{{\text{X}}, 0}} \cdot \exp \left( {\mu \cdot t} \right) \to \ln \left( {\frac{{C_{{\text{X}}} }}{{C_{{{\text{X}}, 0}} }}} \right) = \mu \cdot t,$$

where the sub-index “*F*” refers to the final experimental time (h).

#### Kinetic modelling: simplified reaction network

The methodology developed to design the kinetic model proposed was described elsewhere for anaerobic bioprocesses [38, 39]. The kinetic model is represented in Table 6, according to Eqs. 2–5. The parameters “*ʋ*_*i*/*j*_” represent the empirical yield of the product “*i*” with respect to the substrate “*j*”.

#### Kinetic equations of the model reactions

The kinetic equations of the proposed model are presented in Table 6, through Eqs. 6–9. All have been design according to the mass balances (see also Eqs. 10–18) [37].

#### Calculation of kinetic parameters by fitting to experimental results

The model was fitted to the experimental data using Aspen Custom Modeler (AspenTech, USA), considering six different responses: biomass, CO, acetate, PHB, hydrogen and CO_2_. Model parameters were estimated by minimizing the difference between experimental observations and model simulation according to “*least squares method*” by an adaptive non-linear least-squares algorithm (NL2SOL) [40]. Differential equations were integrated using an implicit Euler method, as previously described elsewhere [39, 41].

The validation of the fittings was performed based on of physicochemical and statistical criteria, including *F*-test (*F*) for a 95% confidence interval, the sum of squared residuals (SSR), the residual mean squared error (RMSE) and the variation explained (VE) [41, 42]. These parameters are defined according to Eqs. 22–24, respectively:

22 $$F = \frac{{ \sum \nolimits_{i = 1}^{N} \left( {\frac{{y_{{i, {\text{calc}}}} }}{P}} \right)^{2} }}{{ \sum \nolimits_{i = 1}^{N} \frac{{{\text{SSR}}}}{N - P}}},$$

23 $${\text{RMSE}} = \sqrt {\frac{{{\text{SSR}}}}{N - P} } ,$$

24 $${\text{VE}} \left( \% \right) = 100\cdot\left( {1 - \frac{{ \sum \nolimits_{l = 1}^{L} {\text{SSQ}}_{l} }}{{ \sum \nolimits_{l = 1}^{L} {\text{SSQ}}_{{{\text{mean}}_{l} }} }}} \right) ,$$

where *N* is the total number of experimental data, *P* the number of parameters in the model; SSR the squared sum of residues and *y*_*i*, calc_ the calculated values of the variable. SSQ_*l*_ and ${\text{SSQ}}_{{{\text{mean}}_{l} }}$ are defined as follows [41]:

25 $${\text{SSQ}}_{l} = \sum \limits_{i = 1}^{N} \frac{{\left( {y_{i, \exp } - y_{{i, {\text{calc}}}} } \right)^{2} }}{{y_{{i, {\text{calc}}}}^{\gamma l} }},$$

26 $${\text{SSQ}}_{{{\text{mean}}_{l} }} = \sum \limits_{i = 1}^{N} \frac{{\left( {y_{i, \exp } - \overline{y}_{i, \exp } } \right)^{2} }}{{y_{{i, {\text{calc}}}}^{\gamma l} }},$$

being

27 $$\overline{y}_{i, \exp } = \frac{{ \sum \nolimits_{i = 1}^{N} \frac{{y_{i, \exp } }}{{y_{{i, {\text{calc}}}}^{\gamma l/2} }}}}{{ \sum \nolimits_{i = 1}^{N} \frac{1}{{y_{{i, {\text{calc}}}}^{\gamma l/2} }}}},$$

where “*γ*_*j*_” is the heteroscedasticity parameter, by means of the type of error in the measured variable. By default, Aspen Custom Modeler fixes its value at 1.

## Data Availability

All data generated during this work are included in this publication.

## Abbreviations

A: Acetate
*A*_i_: Chromatographic area of “i” compound
CBB: Calvin–Benson–Bassham cycle
CIA-CAA: CO detector and alarm actuator
CODH: Carbon monoxide dehydrogenase
COTR: Carbon monoxide transfer rate
COUR: Carbon monoxide uptake rate
*C*_X_: Biomass concentration
DCO: Dissolved carbon monoxide in the liquid phase
EMCoA: Ethylmalonyl-CoA
*F*: Fisher’s parameter in *F*-test
Fd: Ferredoxin
FIC: Flow indicator controller
GC/MS: Gas chromatography coupled to mass spectrometry
HPLC: High-performance liquid chromatography
*K*_1_: H_2_ and CO_2_ production constant in the kinetic model
*K*_2_: H_2_ and CO_2_ inhibition constant in the kinetic model
*K*_H, i_: Henry’s constant of compound “i”
k_L_a: Volumetric gas–liquid mass transfer coefficient
*K*_P_: PHB production constant in the kinetic model
*K*_X/A_: Acetate uptake constant in the kinetic model
$K_{{{\text{X}}/{\text{CO}}_{2} }}$: CO_2_ uptake constant in the kinetic model
MFCs: Mass flow controllers
NL2SOL: Adaptive non-linear squares algorithm
OD_600_: Optical density measured at 600 nm
PHB: Polyhydroxybutyrate
*p*_i_: Partial pressure of compound “i” in inlet or outlet gas stream
*P*_i_: Productivity of compound “i”
PIC: Pressure indicator controller
PNSB: Purple non-sulphur bacteria
*R*: Ideal gas law constant
*R*_i_: Production or consumption rate of compound “i”
*r*_i_: Chemical reaction in the proposed model
RMSE: Root medium squared error
SSR: Sum of squared residuals
*T*: Temperature
TCA: Tricarboxylic acid cycle
VE: Variation explained
VFAs: Volatile fatty acids
*Y*_*i*__/__*j*_: Experimental product “*i*” yield with respect to substrate “*j*”

## Greek symbols

*µ*: Empirical product “*i*” yield with respect to substrate “*j*”
*ʋ*_*i*__/__*j*_: Growth rate
*γ*_*i*_: Heteroscedasticity parameter in the kinetic model
